# Sex differences in the renin-angiotensin-aldosterone system and its roles in hypertension, cardiovascular, and kidney diseases

**DOI:** 10.3389/fcvm.2023.1198090

**Published:** 2023-06-19

**Authors:** Sarah M. Nwia, Ana Paula O. Leite, Xiao Chun Li, Jia Long Zhuo

**Affiliations:** ^1^ Tulane Hypertension and Renal Center of Excellence, Tulane University School of Medicine, New Orleans, LA, United States; ^2^ Department of Physiology, Tulane University School of Medicine, New Orleans, LA, United States; ^3^ Department of Pharmacology, Tulane University School of Medicine, New Orleans, LA, United States

**Keywords:** cardiovascular, hypertension, kidney, renin-Angiotensin system, sex differences

## Abstract

Cardiovascular disease is a pathology that exhibits well-researched biological sex differences, making it possible for physicians to tailor preventative and therapeutic approaches for various diseases. Hypertension, which is defined as blood pressure greater than 130/80 mmHg, is the primary risk factor for developing coronary artery disease, stroke, and renal failure. Approximately 48% of American men and 43% of American women suffer from hypertension. Epidemiological data suggests that during reproductive years, women have much lower rates of hypertension than men. However, this protective effect disappears after the onset of menopause. Treatment-resistant hypertension affects approximately 10.3 million US adults and is unable to be controlled even after implementing ≥3 antihypertensives with complementary mechanisms. This indicates that other mechanisms responsible for modulating blood pressure are still unclear. Understanding the differences in genetic and hormonal mechanisms that lead to hypertension would allow for sex-specific treatment and an opportunity to improve patient outcomes. Therefore, this invited review will review and discuss recent advances in studying the sex-specific physiological mechanisms that affect the renin-angiotensin system and contribute to blood pressure control. It will also discuss research on sex differences in hypertension management, treatment, and outcomes.

## Introduction

Hypertension, defined as blood pressure greater than 130/80 mmHg, has been firmly established as a primary risk factor associated with cardiovascular disease, stroke, and kidney diseases (1–4). In the United States alone, nearly 48% of American men and 43% of American women suffer from hypertension (2). Currently, most if not all available data from clinical studies in humans have consistently shown that premenopausal women are generally protected from the development of hypertension compared with age-matched men, but the prevalence of hypertension increases drastically in women during postmenopausal years. The mechanisms underlying these sex differences or sex dimorphism in the pathogenesis of hypertension in men vs. women remain incompletely understood. Historically, however, biological, physiological, and clinical research were conducted primarily on male cells, male animal models, and male human subjects, largely based upon the assumption that they are genetically, molecularly, and physiologically identical to their female counterparts (5–7). To further promote biomedical research in sex differences in all physiological and diseased models, the National Institute of Health (NIH) in 2014 began to mandate that all recipients of NIH funding are required to consider sex as biological variables in their experimental approaches to test their hypotheses. This policy has led to an explosion of the research on sex differences or sex dimorphism and the mechanisms involved across the board on the disease development and health outcomes (8).

Although hypertension is a multifactorial medical disorder, the renin-angiotensin-aldosterone system (RAAS) is recognized as one of the most important regulators of basal blood pressure homeostasis and a major contributor in the development of hypertension. This recognition is not only supported by extensive biomedical research in animal models of hypertension, but also by numerous clinical trials using the inhibitors of renin, angiotensin-converting enzyme (ACE), or type 1 angiotensin II (Ang II) receptor (AT_1_) or aldosterone receptor blockers to treat hypertension in human subjects (1–4). However, the RAAS is not only the targets for the development and treatment of hypertension, as many hypertensive patients require dual or multidrug therapy with a diuretic, calcium channel blocker, and an α or β blocker to control their blood pressure. Even then, appropriate >10 million Americans still suffer from resistant hypertension even treated with ≥3 antihypertensive medications with blood pressures persisting above the treatment threshold (1–4). The mechanisms underlying the development of resistant hypertension and the difficulty in treating resistant hypertension remain poorly understood. One of major problems may involve sex differences in the pathogenesis, mechanisms, and treatment of resistant hypertension between aging men and postmenopausal women. Thus, there is an urgent need for further studies of the sex differences in the mechanisms of hypertension and the contributions of the RAAS, which may offer more tailored or precision hypertensive treatments and achieve better therapeutic outcomes.

Against this background, the objective of this invited article is to review and discuss recent advances in studying sex differences or dimorphism in the RAAS and its contributions to the physiological regulation of blood pressure and in the development of hypertension, cardiovascular and kidney diseases. Our emphases will include sex differences in the RAAS and the mechanisms by which sex hormones and the RAAS contribute to normal blood pressure control and the development of hypertension, sex differences in the hypertension treatment and outcomes, as well as potential strategies for sex-specific treatment of resistant hypertension in humans.

## Overview of the localization and roles of the RAAS in cardiovascular and kidney tissues

**Figure 1:**
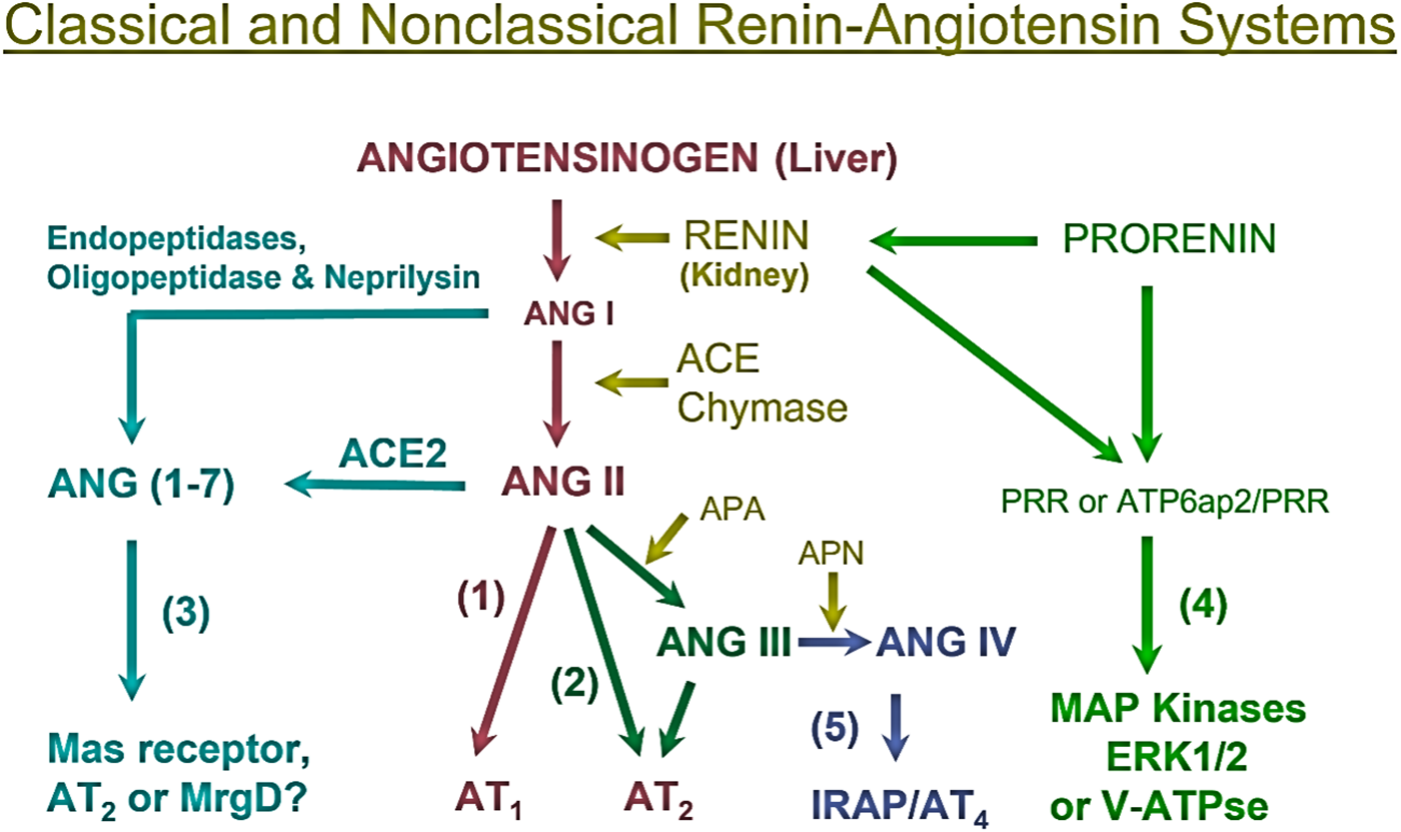
Classical renin/ACE-dependent and non-renin/ACE-dependent pathways for Ang II formation, metabolism, and actions in cardiovascular and kidney tissues. (1) The classical angiotensinogen/renin/ACE/ANG II/AT_1_ receptor axis. (2) The ANG II/APA/ANG III/AT_2_ receptor/NO/cGMP axis. (3) The ANG I/ANG II/ACE2-Neprilysin/ANG (1–7)/*Mas* receptor axis. (4) The prorenin/renin/prorenin receptor (PRR or ATP6ap2)/MAP kinases ERK1/2/V-ATPase axis. (5) The ANG III/APN/ANG IV/AT_4_ receptor/IRAP axis. Note that not only ACE but also chymase generate ANG II from ANG I, whereas neprilysin also cleaves ANG I to generate ANG (1-7). ACE, angiotensin-converting enzyme; ACE2, angiotensin-converting enzyme 2; APA, aminopeptidase A; APN, aminopeptidase N; IRAP, insulin-regulated aminopeptidase; PRR, prorenin receptor. Modified from reference (9) with permission.

To help better understand the sex differences in the RAAS and its contributions to the regulation of cardiovascular and renal physiology and the development of hypertension and cardiovascular and kidney diseases, it is important to first review the localization and roles of the RAAS briefly. The RAAS has been delineated as a primary effector of the development of hypertension and two main axes responsible for blood pressure control have been established. The angiotensinogen (AGT)/renin/angiotensin-converting enzyme (ACE)/angiotensin II (Ang II)/AT_1_ receptor (AGT/renin/ACE/Ang II/AT_1_R) axis is the predominant pathway for Ang II formation and responsible for most if not all classic effects of Ang II in the development of hypertension and cardiovascular and kidney diseases (9) (Figure 1). The juxtaglomerular apparatus of the kidney tightly regulates renin release from the kidney via two important mechanisms—a baroreceptor mechanism that senses decreased blood pressure or blood volume loss within the renal vasculature and an osmoreceptor mechanism that senses NaCl delivery from the proximal nephron to the macula densa (10–14). Renin comprises the rate-limiting step in the activation of the RAAS, converting AGT to Ang I, so its expression levels are in constant balance via a variety of biological mechanisms (15). Ang I is then converted to the biologically active peptide Ang II by ACE. In addition to renin- and ACE-dependent pathways, non-renin/ACE independent pathways may also contribute to the formation and metabolism of Ang II in cardiovascular and kidney tissues (Figure 1). Chymase, a serine endopeptidase, is highly expressed in the heart of patients with cardiovascular diseases compared to ACE (16, 17), and reportedly ∼75% of Ang II is estimated to be generated from Ang (1–8, 10–13) in cardiac tissues by chymase rather than ACE (18, 19). The catalytic activity of chymase is reportedly about 20-fold higher compared to ACE (19, 20). In rats with pressure-overload, the expression of chymase was significantly increased in female than male rats (21). In the kidney, neprilysin (NEP), an endopeptidase, is highly expressed that directly cleaves Ang I into Ang (1-7) and shows much higher catalytic activity for Ang I compared ACE2 (22, 23). The expression of NEP in kidney is reportedly higher in female than male hypertensive mRen (2). Lewis rats (24). Thus, both renin/ACE-dependent and non-renin/ACE-dependent pathways may contribute to Ang II formation or metabolism in cardiovascular and kidney tissues in health and diseases (25, 26) (Figure 1).

**Figure 2:**
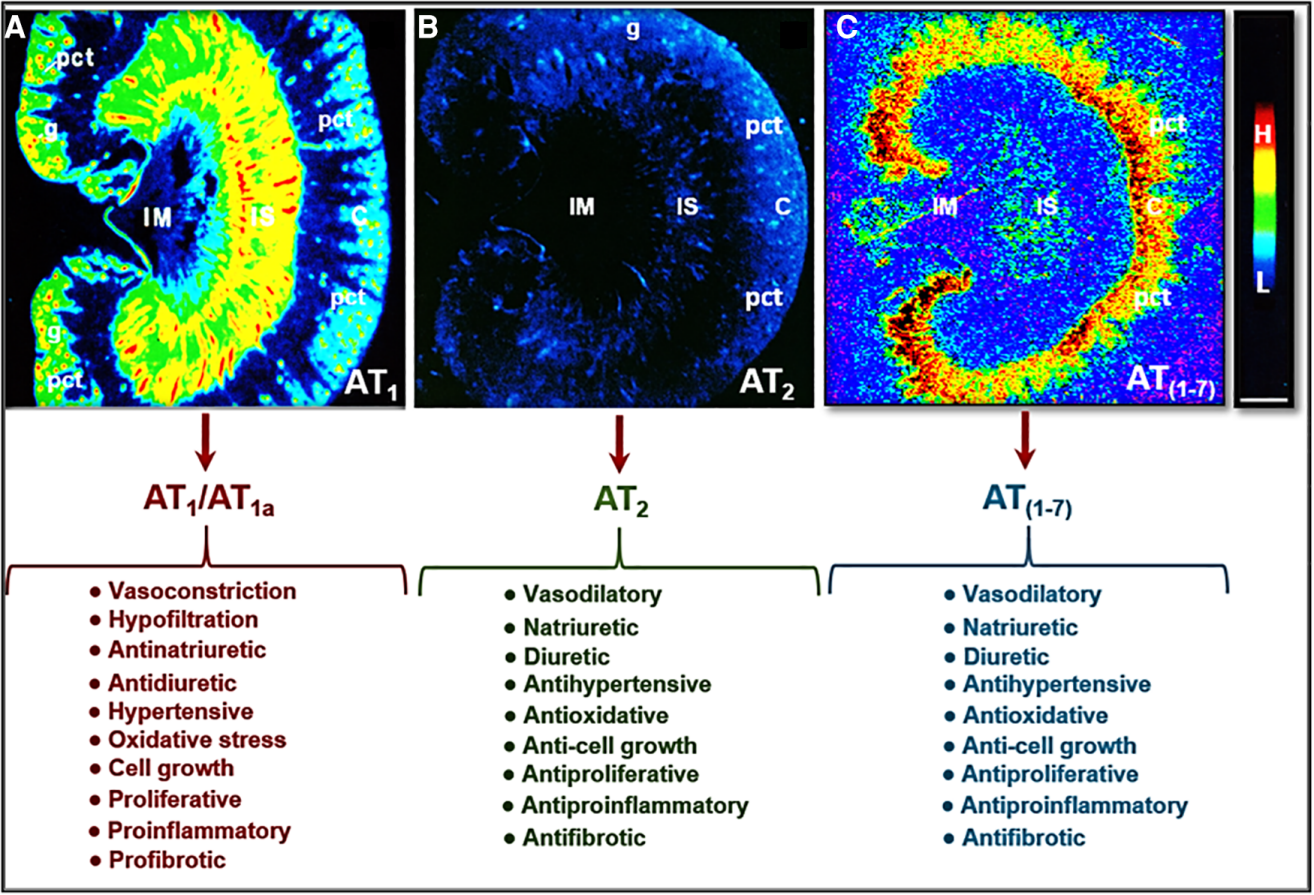
Localization of Ang II type 1 (AT_1_ or AT_1a_) and type 2 receptors (AT_2_) in the rat kidney using ^125^I-labeled Ang II receptor autoradiography and opposing actions of AT_1_ (AT_1a_), AT_2_, and/or AT (1-7) receptor activation in the kidney. (**A**) Shows the localization of AT_1_ or AT_1a_ receptors with high levels in the glomerulus (g) and the inner stripe of the outer medulla corresponding to vasa recta bundles, and moderate levels in the proximal convoluted tubules (pct) in the cortex (C) and renomedullary interstitial cells (RMICs) in the inner stripe of the outer medulla between vasa recta bundles. The inner medulla (IM) expresses a very low level of AT_1_ or AT_1a_. (**B**) Shows the localization of AT_2_ receptors with low levels in the outer cortex, corresponding to the glomeruli and the proximal tubules, and the inner stripe of the outer medulla, corresponding to vasa recta bundles and RMICs. (**C**) Shows the localization of the receptor binding for Ang (1-7) in the kidney primarily in the inner cortex corresponding to the proximal tubules. Red represents high level (H), whereas dark blue represents background levels (L). Modified from reference (30) with permission.

The most pertinent G protein-coupled receptors with which Ang II activates are AT_1_ and AT_2_ receptors. AT_1_ receptors can be classified further into two subtypes: AT_1a_ and AT_1b_. In humans, there is only one AT_1_ receptor that is expressed, corresponding to the AT_1a_ receptor found in rodents (27–29). The AT_1_ receptor is generally considered to have pro-hypertensive, pro-growth, and pro-proliferative downstream effects. Activation of the AT_1_ receptor promotes vasoconstriction, increased oxidative stress, aldosterone release, and renal sodium absorption which all contribute to the regulation of blood pressure and fluid homeostasis, as well as the development of hypertension and cardiovascular and kidney diseases (30, 31) (Figure 2). In the kidney, activation of AT_1_ receptors especially induces the sodium-hydrogen exchanger 3 (NHE3) expression in the proximal tubules and the ascending limp of loop of Henle, resulting in the impairment of the pressure-natriuresis response and an increase in blood pressure (32–36). Conversely, Ang II activation of AT_2_ receptors works against the pro-hypertensive, pro-growth, and proliferative effects of AT_1_ activation, causing vasodilation and increased natriuresis (Figure 2) (34, 37–40). However, Ang III, a biologically active metabolite of Ang II, also acts to increase the natriuresis response reportedly by regulating Na^+^/K^+^-ATPase activity and reducing NHE3 activity (41–44).

**Figure 3:**
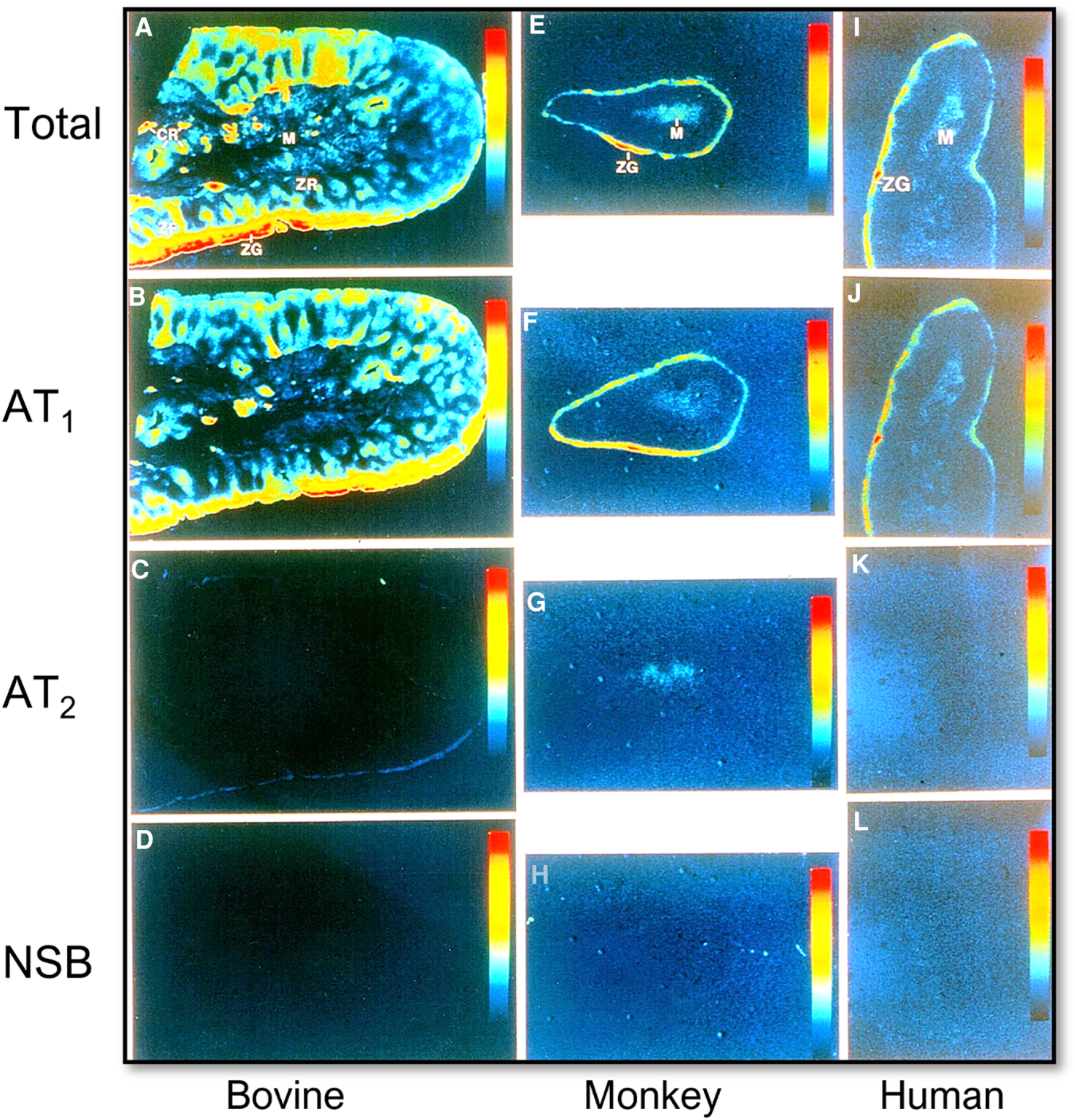
Localization of Ang II type 1 (AT_1_ or AT_1a_) and type 2 receptors (AT_2_) in the bovine, monkey, and human adrenal glands using quantitative ^125^I-labeled Ang II receptor autoradiography. (**A,E,I**) Represent total Ang II receptor binding; (**B,F,J**) represent AT_1_ receptor binding in the presence of an excess concentration of the AT_2_ receptor blocker PD123319 (10 µM); (**C,G,K**) represent AT_2_ receptor binding in the presence of an excess concentration of the AT_1_ receptor blocker losartan (10 µM); and (**D,H,L**) represent nonspecific binding in the presence of an excess concentration of unlabeled Ang II (10 µM), respectively. AT_1_ receptors predominate in the zona glomerulosa cells (ZG) of the adrenal cortex where aldosterone is synthesized and release into the circulation (**B,F,J**), and the adrenal medulla (M). AT_2_ receptors are low in the adrenal glands of bovine, monkey, and human adrenal glands (**C,G,K**). Red represents the highest level, whereas dark blue represents the background level of receptor binding. Modified from reference (32) with permission from the copyright holder.

The final cascade of the RAAS is the release and function of aldosterone from the adrenal glands. Ang II and Ang III both contribute to the stimulation of aldosterone release from the adrenal glands via binding to and activation of AT_1_ and AT_2_ receptors (Figure 3) (45, 46). Aldosterone is a mineralocorticoid that increases blood pressure by inducing the expression and activity of the epithelial sodium channel (ENaC) (47, 48). Previous studies have shown that Ang II stimulates aldosterone secretion in the zona glomerulosa cells (ZG) of the adrenal cortex and catecholamine release from chromaffin cells of the adrenal medulla. The catecholamines may stimulate aldosterone secretion via a paracrine mechanism (49, 50). Most if not all Ang II-induced aldosterone biosynthesis and release from the adrenal glands are mediated by AT_1_ (AT_1a_) receptors. Ang III has been demonstrated to have significant, if not equivocal aldosterone stimulating effects, to Ang II, but is hypothesized to primarily work through AT_2_ receptor activation (46, 51–54). Aldosterone acts to stimulate ENaC expression to increase sodium reabsorption primarily in the distal nephron and collecting tubules, resulting in blood pressure elevation (55). Additionally, increased levels of circulating aldosterone have been found to contribute to the pathogenesis of hypertension by causing endothelial dysfunction via increased production of reactive oxygen species (56).

In addition to the AGT/renin/ACE/Ang II/AT_1_ receptor axis, there exits an alternative counteracting angiotensin-converting enzyme 2 (ACE2)/Ang (1-7)/Mas receptor/AT_2_ receptor (ACE2/Ang (1-7)/MasR/AT_2_R) axis in the cardiovascular and kidney tissues, which is responsible for inducing vasorelaxation, lower blood pressure, and natriuretic responses (9, 57, 58) (Figure 2). Ang (1-7) is a biologically active derivative of Ang I and Ang II that are enzymatically cleaved by ACE2 (57, 58). The primary effects of Ang (1-7) are to counter the effects of the AGT/renin/ACE/Ang II/AT_1_ receptor axis by binding to G-protein coupled Mas receptors (MasR) and inducing the release of nitric oxide (NO), prostaglandin E_2_, and bradykinin to promote vasodilation (59–64). Ang (1-7) infusion was also found to reduce plasma renin activity, which may contribute to its antihypertensive effect (65).

In the kidney, the (pro)renin receptor (PRR) is another receptor that has been established as an important RAAS modulator in the cardiovascular and kidney tissues. PRR is encoded by the ATP6AP2 gene on the X chromosome and has been localized to many tissues including adipose, heart, brain, vessel wall, placenta, and kidney (66–71). Three forms of the protein exist including PRR, soluble PRR (sPRR), and truncated PRR (tPRR). sPRR is released into the plasma, while tPRR remains within the cellular membrane. PRR binds to renin and prorenin resulting in approximately a 5-fold increase in angiotensinogen conversion to angiotensin I (72). PRR has been implicated in both water and sodium homeostasis, as well. During water deprivation trials, PRR and sPRR expression is markedly increased and animal models with principal cell specific PRR deletion have demonstrated significant reductions in AQP2 expression and urine osmolality (73–76). Ang II has also been found to increase AQP2 expression within the collecting duct through several intracellular signaling pathways (77). However, animal studies have demonstrated that chronic Ang II infusion augments sPRR expression which in turn augments water reabsorption via AQP2 demonstrating a positive feedback mechanism within the collecting duct (78). PRR in the collecting duct may cause a marked increase in blood pressure via increasing ENaC expression (76, 79, 80). The precise mechanisms and downstream effects of PRR and its derivatives on water, sodium, and blood pressure have been thoroughly reviewed elsewhere (81).

It is now well-recognized that multiple RAAS axes are working concomitantly to regulate blood pressure and tissue perfusion (32, 34, 43, 82–86). The circulating or classical RAAS including all major components that have well-recognized endocrine effects (15, 32, 34). By contrast, the RAAS in the kidney may represent an important paracrine/autocrine/intracrine system, eliciting a more local and intracellular effect within the kidney tissue, especially within the proximal tubules (32, 34, 43, 82–86). Notably, the intrarenal RAAS has been found to have markedly higher concentrations of Ang II when compared to circulating plasma concentrations (87–93). Chronic Ang II exposure typically causes a down-regulation of AT_1_ receptors in different cardiac and vascular tissues; however, within the intrarenal RAAS, AT_1_ receptor expression is either constant or upregulated during the development of hypertension, cardiovascular and kidney diseases (94, 95).

Recently, there is evidence supporting a functional role for an intracellular and mitochondrial RAS as well. Initial animal studies demonstrated the presence of Ang II binding sites within hepatic cells (33, 92, 96–99). Since then, significant progress has been made in characterizing intracellular RAS within other tissue types. Within the kidney, high-density specific receptors for Ang II and Ang (1-7) were localized to cortical nuclei in sheep and rats (100–103). A fully functional RAS has also been demonstrated within the mitochondria (33, 104, 105). The exact origin of the intracellular RAS and its role in blood pressure homeostasis is yet to be determined, but there is evidence suggesting that they both serve physiological functions in the context of Ang II-induced hypertension (106, 107).

Clearly, recent studies in delineating the vasoconstrictive properties of the AGT/renin/ACE/Ang II/AT_1_ receptor and the vasodilatory properties of the counteracting ACE2/Ang (1-7)/Mas receptor/AT_2_ receptor axes have greatly expanded the therapeutic targets available to treat hypertension and cardiovascular and kidney diseases. Currently, first-line pharmacological treatments for hypertension include monotherapy or combination therapy using ACE inhibitors and angiotensin AT_1_ receptor blockers (ARBs), thiazide diuretics, and long-acting dihydropyridine calcium channel blockers (108, 109). Alpha- and β-blockers have also been identified as adjunctive treatments for hypertension, but they have additional side effects that may make them intolerable to patients including asthma exacerbations, insomnia, worsening glucose intolerance, bradycardia, and sick sinus syndrome (110, 111). Treatment-resistant hypertension is defined as hypertension that is unable to be controlled after the implementation of three antihypertensives with complementary mechanisms (1–4). Now affecting nearly 10.3 million Americans, it has become increasingly prevalent in the United States, indicating a need for alternative or additional therapies (2). Since the classical RAAS has been expanded in recent years, various new drugs have been developed to target these new substrates and receptors. Preclinical data has supported Ang (1-7) and AT_2_ agonists as viable treatment targets, but whether they are effective therapeutic targets in hypertension, cardiovascular and kidney diseases remains to be confirmed in clinical trials (112–114).

## Sex differences in the RAAS and their roles in cardiovascular and renal physiology and hypertension

### Sex differences in vascular dysfunction

Evidence has repeatedly demonstrated that there is an age-dependent difference in the prevalence of hypertension between men and women. Until age 45, women are less likely to develop hypertension than men, while this difference is not present between ages 46 and 64 (2, 115, 116). After age 65, the prevalence among women increases significantly. It is estimated that 85% of women over 75 have hypertension compared with 79% of men within the same age group (2, 115, 116). Recent studies are ongoing to further characterize these differences and underlying mechanisms in the RAAS between males and females, which may contribute to this age-dependent difference in the prevalence of hypertension between men and women.

There are several baseline physiological differences that contribute to the development of hypertension that have been observed in male and female subjects. Nitric oxide (NO), which has vasodilatory effects, has been established as a key mechanism of blood pressure homeostasis (117, 118). NO plays a protective role in the development of hypertension because of its vasodilatory effects and ability to quickly react with superoxide to counteract the latter's effects (119). Animal studies have shown that females have greater NO bioavailability compared with males due to higher NO-generating capacity in females and increased oxidative stress levels in males (120–125). Oxidative stress causes endothelial dysfunction due to vasoconstriction and the activation of the RAAS in blood vessels. *In vivo* studies have shown that Ang II causes mesangial cells in the kidney to produce superoxide, while the inhibition of the RAAS has been shown to reduce oxidative stress (126, 127). More recent data has demonstrated that mice treated with buthionine sulfoximine (BSO), a substance that induces oxidative stress, had higher levels of AT_1_ receptors within the proximal tubules. Additionally, they demonstrated a more dramatic downstream signaling effect, indicating that oxidative stress sensitizes kidney cells to produce an amplified RAS response (128). An inflammatory response to oxidative stress is also activated by Ang II via AT_1_ receptors, leading to nuclear factor-κB (NF-κB) transcription factor expression (128, 129).

## Sex differences and the cardioprotective roles of estrogen

In view of the age differences well-recognized in hypertension prevalence between males and females, the interactions between estrogen and the RAAS have become an important research focus (130). Estrogen is a steroid hormone that binds to two nuclear receptors, estrogen receptor-α (ER-α) and estrogen receptor-β (ER-β), and G protein-coupled estrogen receptor 1 (GPER1) (130–133). ER-α is abundantly expressed in the vascular endothelium and helps promote vasodilation, endothelial repair, and NO production (134). ER-β activation primarily results in NO production (134, 135). Together, the binding of estrogen to these two receptors increases vasodilation and has a protective effect against hypertension. Esqueda et al. demonstrated that after ovariectomy, estrogen-supplemented, salt-sensitive rats had restored ER-β expression levels. The same was not demonstrated for ER-α, implying that the imbalance between ER-α and ER-β might contribute to the development of hypertension after menopause (136).

In animal studies, estradiol has been found to have a role in protecting against hypertension. In spontaneously hypertensive rats (SHRs), young male rats have demonstrated higher mean blood pressures than young female rats (137–140). This difference was eliminated through pharmacological RAS inhibition and the cessation of estrous cycling, implicating estrogen as the cardioprotective factor and accounting for the sex and age-related differences (139, 141, 142). Aging SHRs have been established as a model for postmenopausal hypertension due to their non-cycling, low serum estradiol and the ensuing increase in blood pressure (142, 143).

In human studies, 17β-estradiol (E_2_) has been determined to regulate the RAS via the changes in this enzyme expression. For example, Proudler et al. investigated the effect of estrogen/progesterone combined hormone replacement therapy (HRT) on ACE activity in postmenopausal women. They determined that ACE activity was reduced by 20% in treated women when compared to their untreated controls; however, this study was limited by sample size, including only 28 women in the treatment group and 16 in the untreated group (144). Soon after, Schunkert et al. measured and compared renin and angiotensinogen levels between women treated with estrogen replacement therapy (ERT) and those who were not. Renin levels were found to be significantly increased in women without ERT, measuring 16.6 ± 0.9 mU/L compared to 12.0 ± 0.7 mU/L in the treated group. Angiotensinogen levels were found to be higher in women with ERT, compared to those without, indicating a reduced rate of conversion by renin (145). Thus, these studies provide the evidence for estrogen's cardioprotective effects in part by regulating the expression or activity of the RAS.

## Sex differences in the classical RAS and the role of estrogen

New data has recently built upon these previous studies to elucidate the mechanisms by which estrogen modulates the classical RAS. Essentially, estrogen can alter RAS activities by regulating the levels of key substrate, enzyme, and receptor expression, and protein production. Animal studies have shown that the expression of the RAS enzymes was significantly altered in the presence or absence of estrogen. In young male SHRs, ACE mRNA expression in the kidneys was significantly increased when compared to their female counterparts (146, 147). Similar results were found in two-kidney, one-clip (2K1C) renal hypertension animal models (147). This difference in intratubular enzyme concentrations is attenuated between aging SHR male and female rats (148). In aging SHRs, plasma renin activity (PRA) and concentrations of AGT and Ang II, which are measures of the circulatory RAS activation, were not significantly different between aging male and female SHRs. However, intratubular AGT expression was increased in males when compared to females, whereas aging females were found to have higher Ang II expression (148). These data suggest that in young rats, males have higher levels of intratubular RAS enzyme expression and cascade activation compared to females. In aging rats, when the protective effect of estrogen has diminished, females have increased intrarenal RAS activation and higher levels of Ang II. In addition to the regulation of renin and ACE, estrogen also regulates the renin- and ACE-independent enzymes in the RAS. Ahmad et al. and others compared the metabolic pathway for Ang II formation in cardiac tissues of gonadal-intact and ovariectomized (OVX) adult Wistar Kyoto (WKY) and SHR rats, and found that estrogen depletion significantly increased chymase activity, but not ACE activity (24, 25). Li et al. demonstrated that estrogen inhibits chymase release from cardiac mast cells to prevent pressure overload-induced adverse cardiac remodeling (20). The latter studies suggest that estrogen status may play an important role in the regulation of cardiac chymase expression and cardiovascular protection in adult female animals (20, 24, 25).

Estrogen also plays an important role in regulating the RAS through the modulation of AT_1_ and AT_2_ receptor expression (141). In animal studies comparing arterial AT_1_ expression in male rats, ovariectomized rats, and estrogen-supplemented ovariectomized rats, AT_1_ receptor density was found to be significantly increased in the males and ovariectomized rats when compared to those supplemented with estrogen (140, 149). In aging SHRs, this difference is eliminated and AT_1_ expression was found to be the same between male and female rats (148). Silva-Antonnialli et al. demonstrated that AT_2_ receptor expression was similar among male, female, oophorectomized females, and estrogen-replaced females, causing the AT_1_/AT_2_ ratio in estrogen-treated females to be higher (140). These studies suggest that estrogen's protective role can be partially attributed to its ability to downregulate AT_1_ receptor expression. Indeed, these differences are supported by the studies showing a significant difference in the response to AT_1_ blockers. For instance, aging male rats were observed to have 52% decrease in mean arterial blood pressure, while females only had a 37% drop (148). Increased Ang II or its AT_1_ receptor expression in the kidneys of postmenopausal female rats may explain why postmenopausal women are more susceptible to the development of hypertension and the roles of estrogen in sex differences in hypertension.

The third mechanism by which estrogen can influence blood pressure via the classical RAS is by regulating aldosterone secretion. Aldosterone is known to cause increased salt retention and blood pressure. In animal studies, estrogen was found to reduce AT_1_ receptor expression in the adrenal glands, which in part contribute to reduced aldosterone secretion (150). More recent clinical studies have shown that when consuming high salt diets, men had significantly higher plasma aldosterone, extracellular volume, and systolic blood pressure than women (151). These two studies further suggest that aldosterone secretion may be a key contributor to the sex differences in hypertension prevalence between men and women.

However, the sex differences or the sexual dimorphism of PRR and its role in the development of hypertension remain poorly understood. A study on type 2 diabetic men and women reported that plasma sPRR was significantly higher in women compared to men and that sPRR concentrations appeared to correlate with age, BMI, eGFR, and plasma renin activity in female subjects, though not statistically significant in the male subjects (152). The finding that increased age correlates with increased sPRR and systemic RAS activation suggests that the transition to an estrogen-deficient state of menopause causes increased sPRR expression and RAS activation. However, more work is necessary to characterize the mechanism by which estrogen and PRR interact in further studies.

## Sex differences in the vasoprotective axis of the RAS and the role of estrogen

In addition to inhibitory effects on the classical RAS system, estrogen exerts antihypertensive effects via upregulation of the substrate and enzymes in the counterregulatory RAS pathways. Lee et al. studied ACE2 expression in control and 2K1C male and female rats and demonstrated that female rats showed increased intratubular ACE2 expression regardless of 2K1C treatment status, suggesting estrogen's protective role in increasing Ang II metabolism to Ang (1-7) (147). In studies using human umbilical vein endothelial cells (HUVEC), estrogen activation of ER-α receptors was shown to elevate intracellular ACE and ACE2 mRNA expression and ACE protein expression. This increased ACE2 expression is expected to increase intracellular Ang (1-7) formation (153). This data supports the hypothesis that the intracellular RAS, especially ACE2 and Ang (1-7), and estrogen cooperate in a manner that protects against the development of 2K1C renal hypertension, most likely due to increased Ang (1-7) production and AT_2_ receptor activation.

The MasR is another component of the alternative vasoprotective RAS pathway that demonstrates sex-dependent properties. Previous studies have solidified the hypothesis that NO release is mediated by Ang (1-7) activation of MasR (64, 154, 155). Sobrino et al. used HUVEC to demonstrate that estradiol increased the intracellular expression of enzymes responsible for Ang (1-7) and NO production (156). Their data showed that estradiol treatment increased ACE and cathepsin A expression which are ultimately responsible to produce Ang (1-7). These authors also reported that eNOS and cytosolic guanylate cyclase expression was increased, indicating that NO synthesis was promoted by estradiol treatment. When MasR was blocked, they found that NO levels were decreased, supporting their hypothesis that estradiol mediates increased NO production via the activation of MasR (156). Mompéon et al. also used HUVEC to show that estradiol increased Ang (1-7) production via ER-α activation and increased ACE2 mRNA expression (153). One limitation of these studies, however, is the tissue-specific characteristics of intracellular RAS. It would be beneficial to utilize human or animal kidney cells to fully determine the relationship between estrogen treatment and intracellular RAS responses in the kidney.

In addition to *in vitro* cell culture studies, animal studies have also demonstrated estrogen effects on MasR function. When subjected to Ang II infusion, female rats demonstrated reduced renal blood flow responses, but only in the context of dual MasR and AT_1_ blockade (157, 158). With AT_1_ blockade, there is an increased concentration of circulating Ang II, possibly allowing for increased Ang (1-7) formation via the ACE2 pathway. Saberi et al. compared the effects of estrogen supplementation in response to Ang (1-7) infusion and MasR blockade. They found that estradiol-treated ovariectomized rats had decreased renal blood flow in response to Ang (1-7) after MasR blockade when compared to their untreated counterparts (159). These studies suggest that one of estradiol's antihypertensive mechanisms operates via MasR activation. When MasR is blocked, there are fewer opportunities for estrogen to exert protective effects leading to decreased renal blood flow and worsening hypertension.

Finally, an additional protective axis of the RAAS consisting of Ang III/AT_2_ receptor activation is also modified by estrogen. Female mice have been shown to utilize the AT_2_ receptor pathway to attenuate the effects of Ang II via AT_1_ receptors; however, this effect diminishes with increased age (160, 161). Another study demonstrated that exogenous estrogen replacement reinstituted this protective pathway and attenuated Ang II-induced hypertension (162). Together, these studies support the hypothesis that estrogen affects the RAS primarily through activation of the vasoprotective signaling pathways, rather than the attenuation of the classical RAAS signaling pathway. This evidence could result in novel therapeutics for estrogen-deficient individuals who are suffering from resistant hypertension.

## Sex differences in Ang II-induced hypertension and the roles of testosterone and estrogen

There is no question that testosterone contributes to sex differences in cardiovascular and kidney diseases and hypertension, but its contribution to sex differences is not as well-studied as that of estrogen. Historically, there are animal studies showing mild adverse effects of testosterone on hypertensive outcomes in young spontaneously hypertensive rats (138, 139, 163, 164). Dalmasso et al. have suggested that in aging SHRs, testosterone supplementation causes a reduction of blood pressure, indicating that age, in concordance with testosterone status, affects hypertensive outcomes rather than testosterone alone (164). A more recent animal study determined that testosterone played a permissive role in the development of hypertension since Ang II-induced hypertension was worsened when castrated males were supplemented with exogenous testosterone (165). They also noted that castrated males demonstrated a reduced AT_1_/AT_2_ receptor ratio, which favors the vasoprotective axis of the RAS. This ratio was restored when testosterone was re-administered (165). A mendelian randomization model concluded that high testosterone states could lead to increased rates of hypertension (166). Studies utilizing human subjects present only mildly convincing data. In women specifically, one study showed some evidence that high testosterone states were correlated with increased carotid-femoral pulse wave velocities, which is an indicator of arterial stiffness (167). One review article summarizing the effects of testosterone therapy on various laboratory markers of transgender men concluded that there was only weak evidence supporting the correlation between increased blood pressure and testosterone administration (168). Interestingly, some studies have correlated testosterone-deficient states to the development of hypertension, which would appear to be contrary to the trends observed in previous studies. One such study investigated the effects of free testosterone and biologically available testosterone on blood pressure. It found that free testosterone is essentially inversely correlated with systolic and diastolic blood pressure in men (169). Given the evidence, it is likely that increased testosterone levels in conjunction with decreased estrogen levels, like those found in PCOS, work synergistically to facilitate the development of hypertension. Further research is necessary to characterize the mechanisms by which testosterone regulates blood pressure and its role in the development of hypertension.

Whether there are sex differences in Ang II-dependent or Ang II-induced hypertension remains to be further studied. Some inconsistencies have been reported in the roles of sex differences in Ang II-induced hypertension in animal models (160–162, 170, 171). These inconsistencies range from complete reversal, attenuated responses, or no effect at all in female rats or mice, based on the doses of Ang II infusion (low pressor or high pressor), animal models (rat or mouse, global AT_1a_ or AT_2_ receptor knockout), or routes of administration (subcutaneous or intraperitoneal infusion) (160–162, 170, 171). It is difficult to directly compare these studies and draw a clear conclusion on whether sex differences contribute to the development of Ang II-induced hypertension. Indeed, no significant sex differences in basal blood pressure levels in age-matched adult male and female Sprague-Dawley rats, wild-type, or AT_2_ receptor knockout mice in which Ang II induced similar increases in blood pressure, natriuretic, or diuretic responses (172–175).

**Figure 4:**
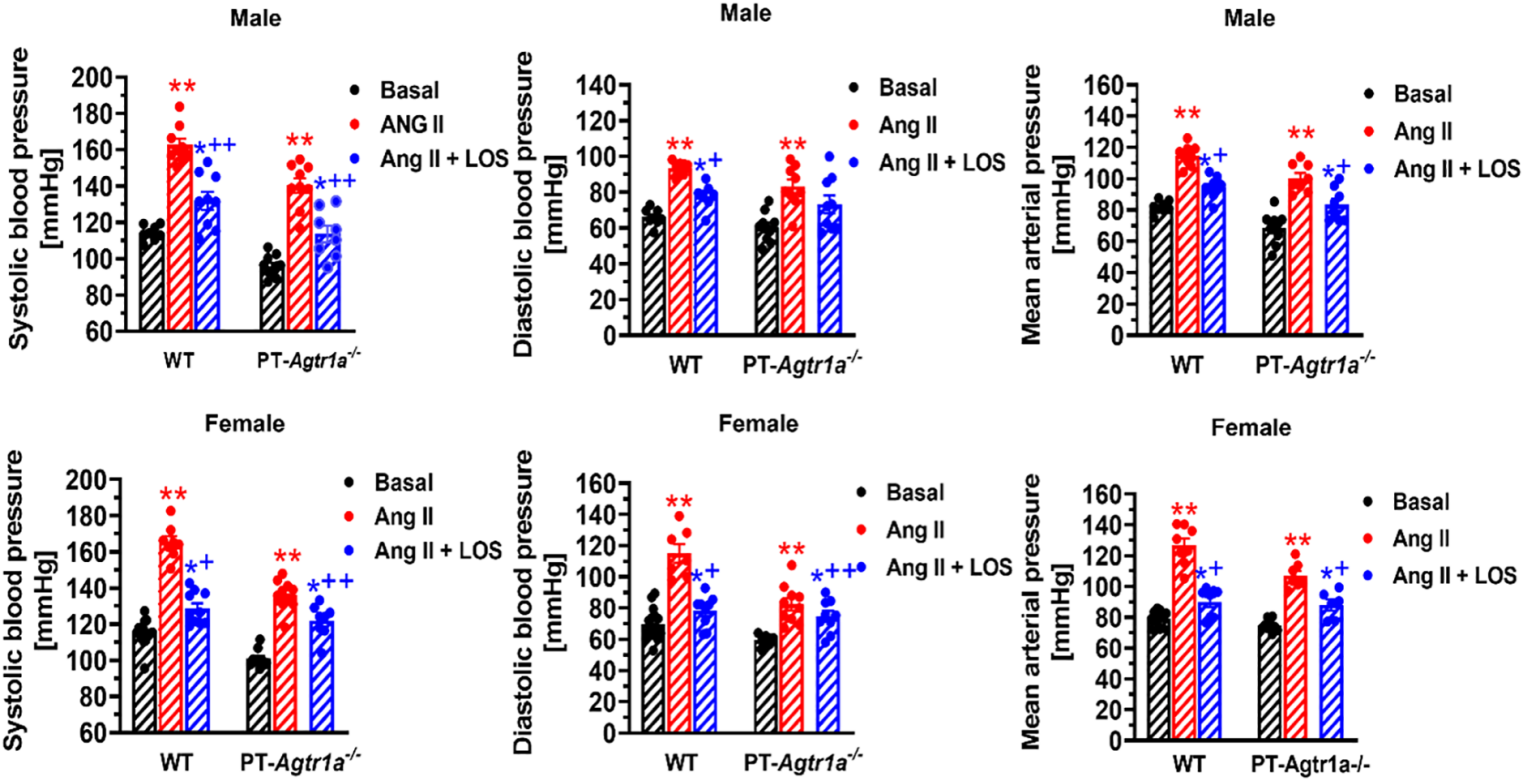
Comparisons of basal systolic, diastolic, and mean arterial blood pressure and their responses to Ang II infusion with or without AT_1_ (AT_1a_) receptor blocker losartan between male and female wild-type (WT) and PT-*Agtr1a^−/−^* mice. Proximal tubule-specific deletion of AT_1a_ receptors significantly decreased basal blood pressure similarly in male and female PT-*Agtr1a^−/−^* mice under basal conditions, and significantly attenuated the hypertensive responses to Ang II similarly in both male and female PT-*Agtr1a^−/−^* mice. No significant sex differences were found in basal blood pressure and its responses to Ang II with or without losartan treatment between male and female WT or between male and female PT-*Agtr1a^−/−^* mice. *P < 0.05 or **P < 0.01 vs. control WT or PT-*Agtr1a^−/−^* mice; ^+^*P *< 0.05 or ^++^*P *< 0.01 vs. Ang II-infused male or female wild-type or PT-*Agtr1a^−/−^* mice. Reproduced from reference (171) with permission.

**Figure 5:**
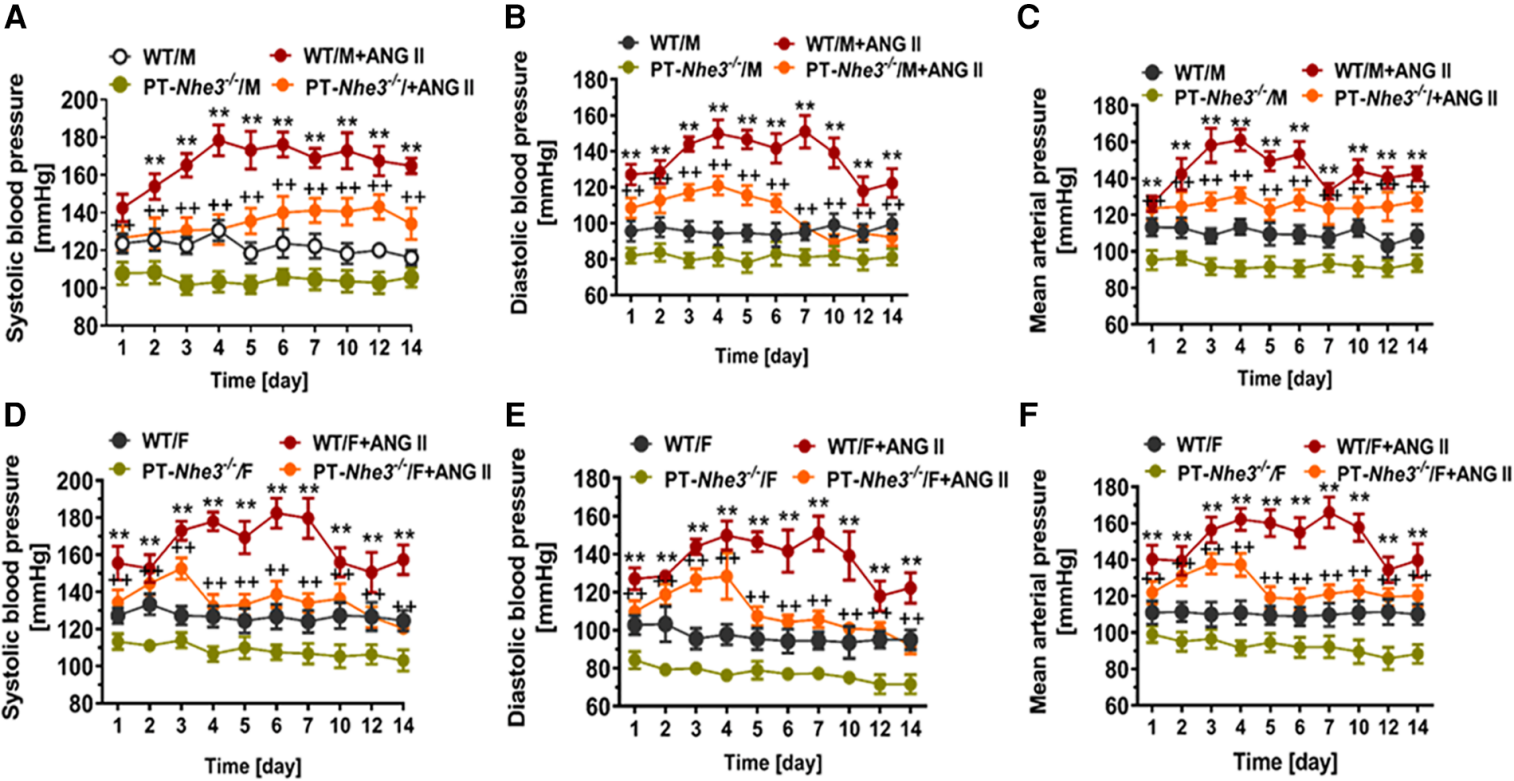
Sex differences in basal systolic, diastolic, and mean arterial blood pressure and their responses to a high pressor dose of Ang II infusion, 1.5 mg/kg per day, intraperitoneal via osmotic minipump in conscious, adult male and female wild-type (WT) and PT-*Nhe3*^−/−^ (proximal tubule-specific NHE3 knockout) mice, as measured using the direct implanted telemetry technique. Please note the time-dependent increases in systolic, diastolic, and mean arterial blood pressure responses to Ang II infusion in male WT mice and significantly attenuated hypertensive responses to Ang II in male PT-*Nhe3*^−/−^ mice. However, systolic, diastolic, and mean arterial blood pressure responses to Ang II began to decrease 4 days after Ang II infusion in female PT-*Nhe3*^−/−^ mice, revealing significant sex differences in these mutant mice. (**A–C**) Male mice; whereas (**D–F**) female mice. **P < 0.01 vs. WT time-control group; ^++^*P *< 0.01 vs. PT-*Nhe3*^−/−^ time-control group, respectively. Reproduced from reference (36) with permission.

Recently, we have determined whether there are sex differences in the blood pressure, renal excretory, and fibrotic responses to Ang II between male and female wild-type mice, and between male and female proximal tubule-specific AT_1a_ receptor knockout mice (PT-*Agtr1a*^−/−^) (170, 171). Although we found sex differences in some minor phenotypic responses, deletion of AT_1a_ receptors selectively in the proximal tubules decreased basal arterial blood pressure similarly in both male and female wild-type and PT-*Agtr1a*^−/−^ mice. Both male and female wild-type and PT-*Agtr1a*^−/−^ mice responded to Ang II infusion and developed hypertension to the similar magnitudes (Figure 4) (170, 171). The maximal pressor responses remained to be ∼20 mmHg lower in male and female PT-*Agtr1a*^−/−^ mice than male and female wild-type mice. Furthermore, concurrent blockade of AT_1_ receptors with losartan decreased the pressor response to Ang II to similar extents in male and female wild-type and PT-*Agtr1a*^−/−^ mice (170, 171). Thus, no significant sexual dimorphism or sex differences in blood pressure phenotypes were discovered in wild-type and PT-*Agtr1a*^−/−^ mice in response to Ang II or AT_1_ receptor blockage. However, we did uncover sex differences in Ang II-induced hypertension in a mutant mouse model with deletion of the Na^+^/H^+^ exchanger 3 (NHE3) selectively in the proximal tubules of the kidney (PT-*Nhe3*^−/−^) (36). In male wild-type and PT-*Nhe3*^−/−^ mice infused with a high pressor dose of Ang II, systolic, diastolic, and mean arterial blood pressure increased in a time-dependent manner reaching a peak response within a week of Ang II infusion (Figure 5). In female PT-*Nhe3*^−/−^ mice, however, systolic, diastolic, and mean arterial blood pressure responses to Ang II began to decrease 4 days after Ang II infusion, suggesting that estrogen (and/or other female hormones) may contribute to these sex differences in Ang II-induced hypertension in this mutant mouse model (Figure 5).

## Sex differences in antihypertensive treatments or managements

In 2017, the American College of Cardiology published new guidelines for the treatment of hypertension. They stratified blood pressure into five categories with different treatment strategies or approaches. Non-pharmacological interventions are an integral part of controlling hypertension of all categories. Lifestyle changes that promote blood pressure reduction include weight loss, DASH diet, sodium intake reduction, dietary potassium supplementation, increased physical activity, and reduced alcohol consumption (1–4). These lifestyle changes are recommended to every patient, regardless of blood pressure status. Patients are initiated on BP-lowering medications once they are diagnosed with Stage 1 and have ASCVD or a 10-year CVD risk ≥10% (1–4). Primary agents for the treatment of hypertension include thiazide diuretics, ACE inhibitors, angiotensin II receptor blockers (ARBs), and calcium channel blockers (CCBs). Secondary agents include loop diuretics, potassium-sparing diuretics, aldosterone antagonists, beta-blockers, direct renin inhibitors, alpha-blockers, and direct vasodilators (1–4).

The INTERHEART study established that elevated blood pressures presented an increased risk for adverse cardiac events for female subjects when compared to male subjects (176). Regarding control, there has been an ongoing debate about the risks and benefits of intensive vs. less intensive therapy. The 2021 SPRINT trial concluded that patients with increased cardiovascular risk were less likely to experience a major adverse cardiac event when their target systolic blood pressure was <120 mmHg when compared to the less intensive <140 mmHg target that was previously established by clinical guidelines (177). When the data is analyzed by sex, the hazard ratio is not statistically significant in the female subgroup. It is important to note that this outcome could be attributed to small female sample size within the trial and lower baseline cardiovascular risk (177). Although the data on blood pressure control is not unanimous, it is generally accepted in clinical practice that a more intensive approach to BP control yields better long-term outcomes (178). Indeed, a study examining worldwide rates of hypertensive control found that blood pressure control rates were significantly worse in women (34.0%) when compared to men (37.7%) (179).

However, current guidelines still do not have sex-specific recommendations when it comes to hypertension management, with an exception for women who are pregnant, breastfeeding, or of childbearing age. One meta-analysis comparing the treatment benefits of ACE inhibitors, CCBs, ARBs, and diuretics/beta-blockers concluded that these blood pressure-lowering regimens all have similar protection against major cardiovascular events between men and women (180). Another study determined that women who have been prescribed losartan were more likely to be hospitalized for angina than their male counterparts receiving the same treatment (181). The ACCOMPLISH trial compared multidrug therapy consisting of ACE inhibitors + CCBs to ACE inhibitors + HCTZ. Their data demonstrated that the ACEI + CCB combination was more effective in reducing adverse cardiovascular events and death, but this same significance was not demonstrated in the female subject subgroup. These findings were likely limited by the fact that only 39.5% of study subjects were women (182). Generally, data demonstrating the relationship between specific antihypertensive regimens and cardiovascular outcomes is lacking when it comes to comparing female and male subjects.

Sex differences have been identified in drug bioavailability, an important factor when it comes to dosing considerations. Women generally have higher gastric pH, slower gastric emptying, and longer gastrointestinal transit time (183). All these features would promote absorption, causing increased drug absorption in women compared to men. After a drug is absorbed, it is distributed around the body into different compartments which can alter bioavailability. Sex differences in body composition such as higher body fat percentage and decreased plasma volume in females could affect drug availability and create higher levels of lipid-soluble drugs in men and hydrophilic drugs in women. Increased bioavailability usually results in increased risk of adverse outcomes, when not accounted for in dosing regimens.

Adverse outcomes to hypertension treatment are an important consideration when trying to optimize cardiovascular outcomes in patients. Rabi et al. reviewed controlled trials of ACE inhibitors and ARBs and found that only 43% of studies reported sex-specific outcomes (184). A comparative study by Rydberg et al. concluded that women had an increased prevalence of adverse drug reactions to ACEIs, thiazides, diuretics, and potassium-sparing agents. When it comes to ACE inhibitor adverse drug reactions (ADRs), female patients were 1.31 times more likely to report adverse reactions (185). The most reported symptoms in both sexes were cough and angioedema (185). Male subjects were more likely to report adverse drug reactions while taking aldosterone antagonists, with the most common reported reaction being hyperkalemia (186). No statistical difference was found between males and females for ARBS, sulfonamides, and selective beta-blockers in the prevalence of adverse drug reactions (187). Overall, female patients are more likely to experience adverse drug reactions while undergoing treatment for hypertension (187–192).

## Concluding remarks

In summary, hypertension remains a critical area of research due to its prevalence and strong association with adverse cardiovascular events. Historically, female subjects have been excluded from *in vivo* animal experiments and clinical trials in humans, leaving half of the population unaccounted for in health, hypertension, cardiovascular, and kidney research. However, recent efforts have increased our understanding of sex differences in the physiological and pathological development of hypertension.

The data summarized in this review highlights the protective effect of estrogen on hypertension. After menopause, women are more likely to develop hypertension due to decreased estrogen levels. Estrogen exerts inhibitory effects on the classical RAAS while promoting non-classical RAS pathways, resulting in an overall vasodilatory and antihypertensive response. However, the mechanisms through which testosterone influences blood pressure remain unclear, and further research is necessary to elucidate its interaction with the RAAS.

Regarding clinical management, there has been some progress in including female subjects in clinical trials. However, research on the clinical outcomes of female and male subjects on specific antihypertensive regimens remains limited. Female patients have been shown to be more prone to adverse drug reactions while undergoing treatment, likely due to sex differences in pharmacokinetics and pharmacodynamics. As such, hypertension treatment that accounts for biological sex might provide better patient outcomes and fewer adverse drug reactions.

Looking towards the future, sex differences in hypertension, cardiovascular and kidney pathogenesis might provide new opportunities to develop novel therapies that not only suppress the classical AGT/renin/ACE/Ang II/AT_1_ receptor responses, but also restore the vasoprotective axis of the ACE2/Ang (1-7)/MasR/AT_2_ receptor responses. For example, therapies that promote Ang (1-7) binding with MasR or activate AT_2_ receptors might be beneficial for postmenopausal women with poorly controlled hypertension, cardiovascular and kidney diseases. Several clinical trials are currently underway to investigate these as viable treatment targets for hypertension.

In conclusion, while some progresses have been made in studying and understanding sex differences in hypertension, cardiovascular and kidney diseases, further research is necessary to develop more effective and personalized treatments that account for biological sex. Inclusion of female subjects in clinical studies is especially critical to help promote clinical decisions that take into account sex-specific factors in the future.
